# Quantized Sampled-Data Control for T-S Fuzzy System Using Discontinuous LKF Approach

**DOI:** 10.3389/fnins.2019.00372

**Published:** 2019-04-24

**Authors:** Shenquan Wang, Shuaiqi Chen, Wenchengyu Ji, Keping Liu

**Affiliations:** College of Electrical and Electronic Engineering Changchun University of Technology, Changchun, China

**Keywords:** stabilization, T-S fuzzy systems, quantization, sampled-data system, discontinuous LKF approach

## Abstract

In this study, the stability for a class of sampled-data Takagi-Sugeno (T-S) fuzzy systems with state quantization was investigated. Using the discontinuous Lyapunov-Krasoskii functional (LKF) approach and the free-matrix-based integral inequality bounds processing technique, a stability condition with less conservativeness has been obtained, and the controller of the sampled-data T-S fuzzy system with the quantized state has been designed. Furthermore, based on the results, the sampled-data T-S fuzzy system without the state quantization was also discussed, and the required controller constructed. The results of two simulation examples show that both the maximum sampling intervals, with and without the quantized state for T-S fuzzy systems, are actually superior to the existing results.

## 1. Introduction

In the real world, most physical systems and processes can be modeled mathematically as complex nonlinear systems. Because some parts of nonlinear systems are always coupled and influence each other, it is difficult to analyze and synthesize these systems. Therefore, establishing an effective and suitable control model to address this issue in nonlinear systems, is significant. In recent years, T-S fuzzy systems has been an effective method to analyze and synthesize nonlinear systems, because of the fact that the T-S fuzzy model is able to transform a complex nonlinear system into several simple linear systems with membership functions, approximating the nonlinear function smoothly with an arbitrary precision in the closed set space, which is ubiquitous in chemical processes, robotics systems, and automatic systems (Tanaka and Wang, 2004). Thus, many results on T-S fuzzy systems, analyzing nonlinear systems, have been reported in terms of various methods, including stability analysis (Lam et al., 2007; Zhao et al., 2009; Zhu et al., 2012, 2018a), controller design (Xia et al., 2010; Wu et al., 2014a,b, 2015; Liu et al., 2016; Wang et al., 2018a; Zhong et al., 2018; Zhao et al., 2019), and fault detection and filter design (Li et al., 2015; Wang et al., 2016, 2017, 2018b; Zhu et al., 2018b), etc.

At the front line of other research, there has been increasing interest in the sampled-data control system, a rapid development of digital and communication technology (Chen and Francis, 2012). The sampled-data control systems involve both continuous-time and discrete-time signals, which make the analysis and synthesis more complicated and challenging. It should be noted that the sampling period is an important issue when analyzing system stability. A larger sampling period can reduce the occupation of the communication channel, and the actuation of the controller and signal transmission. Thus, it is very important to guarantee the stability of the sampled-data system, with a sampling period as large as possible. Over the past several years, sampled-data control systems have drawn much attention (see Hu et al., 2007; Zhu et al., 2012; Shao et al., 2014; Wu et al., 2014a,b, 2015; Liu et al., 2016; Lee and Park, 2017; Wang et al., 2018a and the references therein), and one popular and widely used approach to analyze and synthesize sampled-data systems is the input delay approach, which is based on the representation of the sampled-data system as a continuous-time system with a delayed control input, and does not require the sampling interval to be constant (Fridman et al., 2004; Fridman, 2010; Yang et al., 2014; Li et al., 2015). The authors in Hu et al. (2007) designed a sampled-data controller for networked linear control, and Shao et al. (2014) researched the problem of sampling-interval-dependent stability for sampled-data systems with state quantization. Nevertheless, the results of Hu et al. (2007) and Shao et al. (2014) required the research system to be linear, which is impractical for many applications. Very recently, the problem of stability and stabilization for sampled-data nonlinear fuzzy systems with state quantization was investigated in Liu et al. (2016). Furthermore, the authors in Wang et al. (2018a), designed a dissipativity-based reliable controller for the sampled-data T-S fuzzy system, using the limited Bessel-Legendre inequality proposed in Liu et al. (2017), and a less conservative sufficient stabilization criterion was obtained to guarantee that the sampled-data systems are asymptotically stable. Although there are few results to study the stability of sampled-data T-S fuzzy systems, it is still necessary to decrease the conservativeness of the stability criteria further.

It is well-known that due to the limited capacity and energy consumption in the network system, it is especially important to quantize the sampled-data before transmission. However, most existing studies assume that the data transmission can be performed with infinite precision, and the impact of quantization is always ignored in the network environment. On the other hand, sampling quantization before signal transmission may lead to a limited cycle and chaos. Thus, the study of sampled-data systems with state quantization has attracted significant research attention (Fu and Xie, 2005; Niu et al., 2009; Shao et al., 2014; Liu et al., 2016; Dong et al., 2017a,b). The author in Dong et al. (2017b) studied the reliable control problem for fuzzy systems with state quantization and switched actuator failures. The output feedback control problem of the network control systems with signal quantization and packet loss has been studied in Niu et al. (2009). The stability for sampled-data systems and linear systems with state quantization, have been studied in Dong et al. (2017a), Shao et al. (2014), and Fu and Xie (2005), respectively. Up to now, to the best of authors' knowledge, little attention is given to the issue of quantized sampled-data control for T-S fuzzy systems. Thus, the aim of this work, with a focus on the stability and stabilization problem for sampled-data T-S fuzzy systems with state quantization, is to decrease the conservativeness of the stability criteria even further.

Motivated by that, this work mainly discusses the stability and stabilization control problem for nonlinear T-S fuzzy sampled-data systems with quantized states. By using the discontinuous LKF approach and free-matrix-based integral inequality boundary processing technique, stability conditions with less conservativeness are obtained for T-S fuzzy systems, with sampled-data and quantized states, and the controllers are designed accordingly. Furthermore, the stability of T-S fuzzy sampled-data systems without quantized states is also analyzed utilizing the above theoretical results, and the required sampling data controllers are designed simultaneously. The main contributions of this paper can be summarized as follows: (1) In constructing LKF aspects, it is not necessary that the discontinuous LKF approach applied in this work are positive for all time *t*, only at the sampling times *t*_*k*_ and *t*_*k*+1_, which can broaden the restriction in LKF. (2) In estimating the derivation of LKF, the free-matrix-based integral inequality boundary processing technique is used to provide more freedom in deriving stability for sampled-date T-S fuzzy systems. From two points of view, the conservativeness of stability conditions can be decreased for sampled-data T-S fuzzy systems with and without quantized states effectively through the methods of our design in this work.

***Notations*. **Throughout this paper, *I* denotes the identity matrix with appropriate dimensions. Sym(*A*) denotes *A* + *A*^*T*^. $R^{n}$ denotes the *n* dimensional Euclidean space, and $R^{n\times m}$ is the set of all *n* × *m* real matrices. $S_{+}^{n}$ denotes the *n* dimensional symmetric positive matrices. * denotes the elements below the main diagonal of a symmetric block matrix.

## 2. Problem Formulation

A class of continuous-time nonlinear systems can be described using the following T-S fuzzy model

The rules of plant *i*: If θ_1_(*t*) is *M*_*i*1_(*t*) and … and θ_*n*_(*t*) is *M*_*in*_(*t*), then

(1) $$\begin{array}{r} ẋ\left(t\right)=A_{i}x\left(t\right)+B_{i}u\left(t\right) \end{array}$$

where $x\left(t\right)\in R^{n}$ and $u\left(t\right)\in R^{m}$ are the state and control input vectors of the system, respectively. *A*_*i*_ and *B*_*i*_ are any matrices with appropriate dimensions. θ(*t*) = [θ_1_(*t*)⋯θ_*n*_(*t*)] is the premise variable; *M*_*ij*_(*t*) is the fuzzy set, with *i* = 1, 2, ⋯ , *r* as the amount of fuzzy rules. Then the above continuous time T-S fuzzy system (Equation 1) can be expressed in the following form

(2) $$\begin{array}{r} ẋ\left(t\right)=\overset{r}{\underset{i=1}{\sum }}h_{i}\left(\theta \left(t\right)\right)\left[A_{i}x\left(t\right)+B_{i}u\left(t\right)\right] \end{array}$$

where *h*_*i*_(θ(*t*)) represents the membership function and satisfies $h_{i}\left(\theta \left(t\right)\right)=\omega_{i}\left(\theta \left(t\right)\right)/\overset{r}{\underset{i=1}{\sum }}\omega_{i}\left(\theta \left(t\right)\right)$; $\omega_{i}\left(\theta \left(t\right)\right)=\prod_{j=1}^{n}M_{ij}\left(\theta_{j}\left(t\right)\right)$, where *M*_*ij*_(θ_*j*_(*t*)) is the degree of membership and θ_*j*_(*t*) belongs to the fuzzy set of *M*_*ij*_, which has the following properties. For θ(*t*), ω_*i*_(θ(*t*)) ≥ 0 and $\overset{r}{\underset{i=1}{\sum }}\omega_{i}\left(\theta \left(t\right)\right)>0$, we can determine *h*_*i*_(θ(*t*)) ≥ 0 (∀*i* = 1, 2, ⋯ , *r*) and $\overset{r}{\underset{i=1}{\sum }}h_{i}\left(\theta \left(t\right)\right)=1$.

Suppose the control signal is a sequence of holding times generated by a zero-order-holder function.

$$\begin{array}{l} 0=t_{0}<t_{1}<\cdots <\underset{k\to \infty }{\lim}t_{k}=+\infty \end{array}$$

This paper designs the controller based on the state feedback for T-S fuzzy systems described in (1) and uses the idea of parallel distributed compensation to share the same fuzzy set with the fuzzy model in the premise part of the designed fuzzy controller.

Controller rules *j*: If θ_1_(*t*_*k*_) is *M*_*j*1_(*t*) and … and θ_*n*_(*t*_*k*_) is *M*_*jn*_(*t*), then

(3) $$\begin{array}{r} u\left(t\right)=K_{j}x\left(t_{k}\right),\;t_{k}\leq t\leq t_{k+1},\;j=1,2,\cdots \;,r \end{array}$$

where *K*_*j*_ is the state feedback gain matrix with appropriate dimension, and *x*(*t*_*k*_) is the discrete measurement value at the sampling time of *t*_*k*_.

The logarithm quantizer is described as

$$q\left(•\right)={\left[q_{1}\left(•\right)\;q_{2}\left(•\right)\;\cdots \;q_{n}\left(•\right)\right]}^{T}$$

The *m*th sub-quantizer *q*_*m*_(•) is symmetric, and therefore we have

$$q_{m}\left(x_{m}\left(t_{k}\right)\right)=-q_{m}\left(-x_{m}\left(t_{k}\right)\right)$$

The quantizer satisfies the following quantization criteria

$$\left\{\pm \sigma_{m}^{r}|\sigma_{m}^{r}={\left(\rho_{m}\right)}^{r}\sigma_{m}^{\left(0\right)}\;,\;m=0,\pm 1,\pm 2,\cdots \right\}⋃\left\{0\right\}$$

$$0\leq \rho_{m}<1\;,\;\sigma_{m}^{\left(0\right)}>0$$

where ρ_*m*_ and $\sigma_{m}^{\left(0\right)}$ are quantizer density and initial quantization values, respectively. *q*_*m*_(•) is strictly defined as follows

$$q_{m}(x(t_{k}))=\{\begin{array}{ll} \sigma_{m}^{\left(r\right)},\frac{\sigma_{m}^{\left(r\right)}}{1+l_{m}}\leq x_{m}(t_{k})\leq \frac{\sigma_{m}^{\left(r\right)}}{1-l_{m}}, & if\;x_{m}(t_{k})>0\\ 0, & if\;x_{m}(t_{k})=0\\ -q_{m}(-x_{m}(t_{k})), & if\;x_{m}(t_{k})<0 \end{array}$$

where *l*_*m*_ = 1 − ρ_*m*_/1 + ρ_*m*_(*m* = 1, 2, ⋯ , *n*) is the parameter of the quantizer. When *x*_*m*_(*t*_*k*_) > 0, the following relationship is established

$$\left(1-l_{m}\right)x_{m}\left(t_{k}\right)\leq \sigma_{m}^{\left(r\right)}\leq \left(1+l_{m}\right)x_{m}\left(t_{k}\right)$$

For *x*_*m*_(*t*_*k*_) < 0, the following is satisfied

$$\left(1+l_{m}\right)x_{m}\left(t_{k}\right)\leq \sigma_{m}^{\left(r\right)}\leq \left(1-l_{m}\right)x_{m}\left(t_{k}\right)$$

Therefore, the quantizer can be expressed as

$$q\left(x\left(t_{k}\right)\right)=x\left(t_{k}\right)+f\left(x\left(t_{k}\right)\right)$$

where

$$f\left(x\left(t_{k}\right)\right)={\left[f_{1}\left(x\left(t_{k}\right)\right)\;f_{2}\left(x\left(t_{k}\right)\right)\;\cdots \;f_{n}\left(x\left(t_{k}\right)\right)\right]}^{T}$$

(4) $$\begin{array}{r} -l_{m}{\left[x_{m}\left(t_{k}\right)\right]}^{2}\leq x_{m}\left(t_{k}\right)f_{m}\left(x\left(t_{k}\right)\right)\leq l_{m}{\left[x_{m}\left(t_{k}\right)\right]}^{2} \end{array}$$

Hence, the overall state feedback controller with the quantized state can be designed

(5) $$\begin{array}{r} u\left(t\right)=\overset{r}{\underset{j=1}{\sum }}h_{j}\left(\theta \left(t_{k}\right)\right)K_{j}\left[x\left(t_{k}\right)+f\left(x\left(t_{k}\right)\right)\right] \end{array}$$

Suppose the distance between two consecutive sampling instants belongs to an interval, and then for all *k* > 0 there exists

(6) $$\begin{array}{r} t_{k+1}-t_{k}=h_{k},h_{k}\in \left[h_{L},h_{U}\right] \end{array}$$

where *h*_*L*_ and *h*_*U*_ are known constants satisfying 0 ≤ *h*_*L*_ ≤ *h*_*U*_. Combining Equations (1)–(6), the T-S fuzzy sampled-data system with state quantization can be obtained as follows

(7) $$x(t)=\sum_{i=1}^{r}\;=\sum_{j=1}^{r}h_{i}(\theta (t))h_{j}(\theta (t_{k}))[A_{i}x(t)+B_{i}K_{j}(x(t_{k})+f(x(t_{k})))]$$

The following lemma is important for further analysis.

**Lemma 1**. Lee and Park (2017) (Free-Matrix-Based Integral Inequality) For given matrices $R\in S_{+}^{n}$, *N*_1_, $N_{2}\in R^{3n\times n}$, the following inequality holds for any continuously differentiable function *x*(*t*) in $\left[a,b\right]\in R^{n}$

$$-{\int^{\text{​}}}_{\beta }^{\alpha }{\dot{x}}^{T}(s)R\dot{x}(s)ds\leq \phi^{T}(\alpha ,\beta )\Psi (\alpha ,\beta )\phi (\alpha ,\beta )$$

where

$$\text{Ψ}\left(\alpha ,\beta \right)=\left(\beta -\alpha \right)\left(N_{1}R^{-1}N_{1}^{T}+\frac{{\left(\beta -\alpha \right)}^{2}}{3}N_{2}R^{-1}N_{2}^{T}+Z_{1}\right)+Z_{2}\;$$

$$\phi \left(\alpha ,\beta \right)={\left[\begin{array}{ccc} x^{T}\left(\beta \right) & x^{T}\left(\alpha \right) & \int_{\alpha }^{\beta }x^{T}\left(s\right)ds \end{array}\right]}^{T}$$

$$Z_{1}=-\text{Sym}\left[\begin{array}{ccc} N_{2} & N_{2} & 0 \end{array}\right]$$

$$Z_{2}=\text{Sym}\left[\begin{array}{ccc} N_{1} & -N_{1} & 2N_{2} \end{array}\right]$$

**Remark 1**. Note that the free-matrix-based integral inequality in Lemma 1 provides more freedom in deriving stability for sampled-date T-S fuzzy systems. So, it provides the possibility of finding a tight bound for $-\int_{\alpha }^{\beta }{ẋ}^{T}\left(s\right)Rẋ\left(s\right)ds$. On the other hand, the augmented vector ϕ(α, β) includes $\int_{\alpha }^{\beta }x^{T}\left(s\right)ds$ instead of $\frac{1}{\beta -\alpha }\int_{\alpha }^{\beta }x^{T}\left(s\right)ds$, and this avoids appearance of the term (*t* − *t*_*k*_)ϕ(*t*_*k*_, *t*) in sampled-data systems, which is actually much easier to handle.

## 3. Main Result

### 3.1. Stability Analysis of the Sampled-Data T-S Fuzzy System With State Quantization

In this section, the asymptotic stability conditions of T-S fuzzy systems with state quantization are analyzed.

For the convenience of system analysis and design, we define

$$\begin{array}{lll} e_{i} & = & \left[\begin{array}{ccc} 0_{n\times \left(i-1\right)n} & I_{n} & 0_{n\times \left(5-i\right)n} \end{array}\right],\\ \xi^{T}\left(t\right) & = & \left[\begin{array}{ccccc} x^{T}\left(t\right) & x^{T}\left(t_{k}\right) & \int_{t_{k}}^{t}x^{T}\left(s\right)ds & {ẋ}^{T}\left(t\right) & f^{T}\left(x\left(t_{k}\right)\right) \end{array}\right] \end{array}$$

where *e*_*i*_ is defined as the block entry matrix. Other notations are defined as

$$\begin{array}{lll} \eta_{1}^{T}\left(t\right) & = & \left[\begin{array}{ccc} x^{T}\left(t\right)-x^{T}\left(t_{k}\right) & \int_{t_{k}}^{t}x^{T}\left(s\right)ds & f^{T}\left(x\left(t_{k}\right)\right) \end{array}\right]\\ \eta_{2}^{T}\left(t\right) & = & \left[\begin{array}{ccc} x^{T}\left(t_{k}\right) & {ẋ}^{T}\left(t\right) & f^{T}\left(x\left(t_{k}\right)\right) \end{array}\right]\\ \eta_{3}^{T}\left(t\right) & = & \left[\begin{array}{ccc} {ẋ}^{T}\left(t\right) & x^{T}\left(t\right) & 0 \end{array}\right]\\ \eta_{4}^{T}\left(t\right) & = & \left[\begin{array}{ccc} x^{T}\left(t\right) & x^{T}\left(t_{k}\right) & \int_{t_{k}}^{t}x^{T}\left(s\right)ds \end{array}\right]\\ \eta_{5}^{T}\left(t\right) & = & \left[\begin{array}{ccc} {ẋ}^{T}\left(t\right) & 0 & x^{T}\left(t\right) \end{array}\right]\\ \Pi_{1}^{T} & = & \left[\begin{array}{ccc} e_{1}^{T}-e_{2}^{T} & e_{3}^{T} & e_{5}^{T} \end{array}\right]\\ \Pi_{2}^{T} & = & \left[\begin{array}{ccc} e_{2}^{T} & e_{4}^{T} & e_{5}^{T} \end{array}\right]\\ \Pi_{3}^{T} & = & \left[\begin{array}{ccc} e_{4}^{T} & e_{1}^{T} & 0 \end{array}\right]\\ \Pi_{4}^{T} & = & \left[\begin{array}{ccc} e_{2}^{T} & e_{2}^{T} & e_{3}^{T} \end{array}\right]\\ \Pi_{5}^{T} & = & \left[\begin{array}{ccc} e_{4}^{T} & 0 & e_{1}^{T} \end{array}\right] \end{array}$$

**Theorem 1**. For given scalars *h*_*L*_ and *h*_*U*_, satisfying 0 ≤ *h*_*L*_ ≤ *h*_*U*_, system (Equation 7) is asymptotically stable, if there are some symmetric positive definite matrices $P\in S_{+}^{n}$, *X*_1_, $Q\in S_{+}^{3n}$, $R\in S_{+}^{7n}$; a diagonal matrix $D\in S_{+}^{n}$; any matrices *G*_1_, *G*_2_, *X*_2_, *N*_1_, *N*_2_ with appropriate dimensions, and for any *i* ≥ 1 and *j* ≤ *r*, the following linear matrix inequalities (LMIs) hold

(8) $$\begin{array}{r} \Phi_{1}+h_{k}\Phi_{2}<0 \end{array}$$

(9) $$\begin{array}{r} \left[\begin{array}{ccc} \Phi_{1}+h_{k}\Phi_{3} & \Phi_{12} & \Phi_{13}\\ {}* & -Q_{4} & 0\\ {}* & * & -3Q_{4} \end{array}\right]<0 \end{array}$$

where

$$\begin{array}{l} \Phi_{1}=\text{Sym}\left(e_{1}^{T}Pe_{4}\right)-\Pi_{1}^{T}X_{1}\Pi_{1}-\text{Sym}\left(e_{3}^{T}X_{2}e_{2}\right)\\ -\text{Sym}\left({\left[\begin{array}{c} e_{2}\\ e_{5} \end{array}\right]}^{T}\left[\begin{array}{c} Q_{2}\\ Q_{5} \end{array}\right]\left(e_{1}-e_{2}\right)\right)+\Pi_{4}^{T}{ℜ}_{1}\Pi_{4}\\ \;+\text{Sym}\left(\Pi_{4}^{T}{ℜ}_{2}\Pi_{5}\right)+e_{4}^{T}R_{6}e_{4}+\Pi_{4}^{T}Z_{2}\Pi_{4}\\ \;+\text{Sym}\left(\left(e_{1}^{T}G_{1}+e_{4}^{T}G_{2}\right)\text{Ψ}\right)\\ \;-\text{Sym}\left[{\left(e_{5}+Le_{2}\right)}^{T}D\left(e_{5}-Le_{2}\right)\right] \end{array}$$

$$\Phi_{2}=\text{Sym}\left(\Pi_{1}^{T}X_{1}\Pi_{3}\right)+\text{Sym}\left(e_{1}^{T}X_{2}e_{2}\right)+\Pi_{2}^{T}Q\Pi_{2}$$

$$\Phi_{3}=-{\left[\begin{array}{c} e_{2}\\ e_{5} \end{array}\right]}^{T}\left[\begin{array}{cc} Q_{1} & Q_{3}\\ {}* & Q_{6} \end{array}\right]\left[\begin{array}{c} e_{2}\\ e_{5} \end{array}\right]+\text{Sym}\left(\Pi_{4}^{T}{ℜ}_{1}\Pi_{5}\right)+\Pi_{4}^{T}Z_{1}\Pi_{4}$$

$$\Phi_{12}=\sqrt{h_{k}}\Pi_{4}^{T}N_{1}$$

$$\Phi_{13}=h_{U}\sqrt{h_{k}}\Pi_{4}^{T}N_{2}$$

with

$${ℜ}_{1}=R_{1}+\frac{h_{U}^{2}}{3}R_{4}-\text{Sym}\left[\begin{array}{ccc} R_{5} & R_{5} & 0 \end{array}\right]$$

$${ℜ}_{2}=\text{Sym}\left[\begin{array}{ccc} R_{3} & -R_{3} & 2R_{5} \end{array}\right]$$

$$\text{Ψ}=\left[\begin{array}{ccccc} A_{i} & B_{i}K_{j} & 0 & -I & B_{i}K_{j} \end{array}\right]$$

$$Q=\left[\begin{array}{ccc} Q_{1} & Q_{2} & Q_{3}\\ {}* & Q_{4} & Q_{5}\\ {}* & * & Q_{6} \end{array}\right]$$

$$R=\left[\begin{array}{ccc} R_{1} & R_{2} & R_{3}\\ {}* & R_{4} & R_{5}\\ {}* & * & R_{6} \end{array}\right]$$

and *Z*_1_, *Z*_2_ have been defined in Lemma 1.

Proof: The novel discontinuous LKF in this work is constructed as follows

(10) $$\begin{array}{r} V\left(t\right)=V_{1}\left(t\right)+V_{2}\left(t\right)+V_{3}\left(t\right)\;t\in \left[t_{k},t_{k+1}\right] \end{array}$$

where

$$\begin{array}{l} V_{1}(t)=x^{T}(t)Px(t)\\ V_{2}(t)=(t_{k+1}-t)(\eta_{1}^{T}(t)X_{1}\eta_{1}(t)+Sym\left(\int_{t_{k}}^{t}x^{T}(s)dsX_{2}x(t_{k})\right)\\ \;+\;\int_{t_{k}}^{t}\eta_{2}^{T}(s)Q\eta_{2}(s)ds)\\ V_{3}(t)=\eta_{4}^{T}(t)ℜ\eta_{4}(t)+\int_{t_{k}}^{t}{\dot{x}}^{T}(s)R_{6}\dot{x}(s)ds \end{array}$$

with

$$\begin{array}{l} ℜ=\left(t-t_{k}\right){ℜ}_{1}+{ℜ}_{2} \end{array}$$

**Remark 2**. It should be noted that the constructed LKF (Equation 10) of this work is the discontinuous Lyapunov functional, as *V*_3_(*t*) is actually a time-dependent discontinuous term. Moreover, it is not necessary that *V*_1_(*t*) and *V*_2_(*t*) in LKF (Equation 10) are positive for all time *t*, but only positive at the sampling times *t*_*k*_ and *t*_*k*+1_. All this can decrease the conservativeness of stability conditions effectively.

The derivative of *V*_1_(*t*), *V*_2_(*t*), and *V*_3_(*t*) can, respectively, be deduced as

(11) $$\begin{array}{r} {\overset{•}{V}}_{1}\left(t\right)=2x^{T}\left(t\right)Pẋ\left(t\right) \end{array}$$

(12) $$\begin{array}{l} {\dot{V}}_{2}(t)=-\eta_{1}^{T}(t)X_{1}\eta_{1}(t)-\text{Sym}\left(\int_{t_{k}}^{t}x^{T}(s)dsX_{2}x(t_{k})\right)\\ -\text{Sym}\left({\left[\begin{array}{c} x(t_{k})\\ f(x(t_{k})) \end{array}\right]}^{T}\left[\begin{array}{c} Q_{2}\\ Q_{5} \end{array}\right](x(t)-x(t_{k}))\right)\\ -(t-t_{k})\left[\begin{array}{c} x(t_{k})\\ f(x(t_{k})) \end{array}\right]T\left[\begin{array}{cc} Q_{1} & Q_{3}\\ {}* & Q_{6} \end{array}\right]\left[\begin{array}{c} x(t_{k})\\ f(x(t_{k})) \end{array}\right]\\ \;-\int_{t_{k}}^{t}{\dot{x}}^{T}(s)Q_{4}\dot{x}(s)ds\\ \;+(t_{k+1}-t)(\text{Sym}\left(\eta_{1}^{T}(t)X_{1}\eta_{3}(t)\right)\\ \;+\text{Sym}\left(x^{T}(t)X_{2}x(t_{k})\right)+\eta_{2}^{T}(t)Q\eta_{2}(t)) \end{array}$$

(13) $$\begin{array}{r} {\overset{•}{V}}_{3}\left(t\right)=\eta_{4}^{T}\left(t\right){ℜ}_{1}\eta_{4}\left(t\right)+\text{Sym}\left(\eta_{4}^{T}\left(t\right)ℜ\eta_{5}\left(t\right)\right)+{ẋ}^{T}\left(t\right)R_{6}ẋ\left(t\right) \end{array}$$

By Lemma 1, we obtain the inequality

(14) $$\begin{array}{r} -\int_{t_{k}}^{t}{ẋ}^{T}\left(s\right)Q_{4}ẋ\left(s\right)ds\leq \eta_{4}^{T}\left(t\right)\left(\left(t-t_{k}\right)\left(N_{1}Q_{4}^{-1}N_{1}^{T}+\frac{{\left(t-t_{k}\right)}^{2}}{3}N_{2}Q_{4}^{-1}N_{2}^{T}+Z_{1}\right)+Z_{2}\right)\\ \eta_{4}\left(t\right) \end{array}$$

Based on the closed-loop system (Equation 7), the following equality holds

$$\begin{array}{l} \begin{array}{c} \text{Sym}\left(x^{T}\left(t\right)G_{1}+{ẋ}^{T}\left(t\right)G_{2}\right)\times \\ \left(-ẋ\left(t\right)+\overset{r}{\underset{i=1}{\sum }}\overset{r}{\underset{j=1}{\sum }}h_{i}\left(\theta \left(t\right)\right)h_{j}\left(\theta \left(t_{k}\right)\right)\left[A_{i}x\left(t\right)+B_{i}K_{j}x\left(t_{k}\right)+B_{i}K_{j}f\left(x\left(t_{k}\right)\right)\right]\right)=0, \end{array} \end{array}$$

and it can be further written as

(15) $$\begin{array}{r} \overset{r}{\underset{i=1}{\sum }}\overset{r}{\underset{j=1}{\sum }}h_{i}\left(\theta \left(t\right)\right)h_{j}\left(\theta \left(t_{k}\right)\right)\text{Sym}\left(x^{T}\left(t\right)G_{1}+{ẋ}^{T}\left(t\right)G_{2}\right)\\ \left(\left[\begin{array}{ccccc} A_{i} & B_{i}K_{j} & 0 & -I & B_{i}K_{j} \end{array}\right]\xi \left(t\right)\right)=0 \end{array}$$

From Equation (4), for the diagonal matrix *D* > 0, it holds that

(16) $$\begin{array}{r} -\text{Sym}\left({\left[f\left(x\left(t_{k}\right)\right)+Lx\left(t_{k}\right)\right]}^{T}D\left[f\left(x\left(t_{k}\right)\right)-Lx\left(t_{k}\right)\right]\right)\geq 0 \end{array}$$

Then combining (Equations 11–16), an upper bound of $\dot{V}\left(t\right)$ can be obtained as follows

(17) $$\begin{array}{r} \dot{V}\left(t\right)\leq \overset{r}{\underset{i=1}{\sum }}\overset{r}{\underset{j=1}{\sum }}h_{i}\left(\theta \left(t\right)\right)h_{j}\left(\theta \left(t_{k}\right)\right)\xi^{T}\left(t\right)\\ \left(\frac{t_{k+1}-t}{h_{k}}\left(\Phi_{1}+h_{k}\Phi_{2}\right)+\frac{t-t_{k}}{h_{k}}\left(\Phi_{1}+h_{k}{\overset{}{\overset{⌣}{\Phi }}}_{3}\right)\right)\xi \left(t\right) \end{array}$$

where

$$\begin{array}{l} \begin{array}{c} {\overset{}{\overset{⌣}{\Phi }}}_{3}=-{\left[\begin{array}{c} e_{2}\\ e_{5} \end{array}\right]}^{T}\left[\begin{array}{cc} Q_{1} & Q_{3}\\ {}* & Q_{6} \end{array}\right]\left[\begin{array}{c} e_{2}\\ e_{5} \end{array}\right]+\text{Sym}\left(\Pi_{4}^{T}{ℜ}_{1}e_{2}\right)+\Pi_{4}^{T}Z_{1}\Pi_{4}\\ +\Pi_{4}^{T}\left(N_{1}Q_{4}^{-1}N_{1}^{T}+\frac{{\left(t-t_{k}\right)}^{2}}{3}N_{2}Q_{4}^{-1}N_{2}^{T}\right)\Pi_{4} \end{array} \end{array}$$

with the following two equalities

$$\Phi_{1}+h_{k}\Phi_{2}=\frac{h_{U}-h_{k}}{h_{U}-h_{L}}\left(\Phi_{1}+h_{L}\Phi_{2}\right)+\frac{h_{k}-h_{L}}{h_{U}-h_{L}}\left(\Phi_{1}+h_{U}\Phi_{2}\right)$$

$$\Phi_{1}+h_{k}{\overset{}{\Phi }}_{3}=\frac{h_{U}-h_{k}}{h_{U}-h_{L}}\left(\Phi_{1}+h_{L}{\overset{}{\Phi }}_{3}\right)+\frac{h_{k}-h_{L}}{h_{U}-h_{L}}\left(\Phi_{1}+h_{U}{\overset{}{\Phi }}_{3}\right)$$

Therefore, using Schur Complement, Equations (8) and (9) are equivalent to $\dot{V}\left(t\right)<0$. This completes the proof.

Additionally, Theorem 1 has provided the stability results for T-S fuzzy sampled-data systems (Equation 7) with state quantization, and, the following Theorem 2 will be given in order to obtain the sampled-data controller.

**Theorem 2**. For given scalars *h*_*L*_ and *h*_*U*_, satisfying 0 ≤ *h*_*L*_ ≤ *h*_*U*_, system (Equation 7) is asymptotically stable, if there are some symmetric positive definite matrices $\bar{P}\in S_{+}^{n}$, ${\bar{X}}_{1}$, $\bar{Q}\in S_{+}^{3n}$, $\bar{R}\in S_{+}^{7n}$; diagonal matrix $\bar{D}\in S_{+}^{n}$; any matrices *G*, *T*_*j*_, ${\bar{X}}_{2}$, ${\bar{N}}_{1}$, ${\bar{N}}_{2}$ with appropriate dimensions, and for any *i* ≥ 1 and *j* ≤ *r*, the following LMIs hold

(18) $$\begin{array}{r} {\bar{\Phi }}_{1}+h_{k}{\bar{\Phi }}_{2}<0 \end{array}$$

(19) $$\begin{array}{r} \left[\begin{array}{ccc} {\bar{\Phi }}_{1}+h_{k}{\bar{\Phi }}_{3} & {\bar{\Phi }}_{12} & {\bar{\Phi }}_{13}\\ {}* & -{\bar{Q}}_{4} & 0\\ {}* & * & -3{\bar{Q}}_{4} \end{array}\right]<0 \end{array}$$

where

$$\begin{array}{l} {\bar{\Phi }}_{1}=\text{Sym}\left(e_{1}^{T}\bar{P}e_{4}\right)-\Pi_{1}^{T}{\bar{X}}_{1}\Pi_{1}-\text{Sym}\left(e_{3}^{T}{\bar{X}}_{2}e_{2}\right)\\ \;-\text{Sym}\left({\left[\begin{array}{c} e_{2}\\ e_{5} \end{array}\right]}^{T}\left[\begin{array}{c} {\bar{Q}}_{2}\\ {\bar{Q}}_{5} \end{array}\right]\left(e_{1}-e_{2}\right)\right)+\Pi_{4}^{T}{\bar{ℜ}}_{1}\Pi_{4}\\ \;+\text{Sym}\left(\Pi_{4}^{T}{\bar{ℜ}}_{2}\Pi_{5}\right)+e_{4}^{T}{\bar{R}}_{6}e_{4}+\Pi_{4}^{T}{\bar{Z}}_{2}\Pi_{4}\\ \;+\text{Sym}\left(\left(e_{1}^{T}+\varepsilon e_{4}^{T}\right)\bar{\text{Ψ}}\right)\\ \;-\text{Sym}\left[{\left(e_{5}+Le_{2}\right)}^{T}\bar{D}\left(e_{5}-Le_{2}\right)\right] \end{array}$$

$${\bar{\Phi }}_{2}=\text{Sym}\left(\Pi_{1}^{T}{\bar{X}}_{1}\Pi_{3}\right)+\text{Sym}\left(e_{1}^{T}{\bar{X}}_{2}e_{2}\right)+\Pi_{2}^{T}\bar{Q}\Pi_{2}$$

$${\bar{\Phi }}_{3}=-{\left[\begin{array}{c} e_{2}\\ e_{5} \end{array}\right]}^{T}\left[\begin{array}{cc} {\bar{Q}}_{1} & {\bar{Q}}_{3}\\ {}* & {\bar{Q}}_{6} \end{array}\right]\left[\begin{array}{c} e_{2}\\ e_{5} \end{array}\right]+\text{Sym}\left(\Pi_{4}^{T}{\bar{ℜ}}_{1}\Pi_{5}\right)+\Pi_{4}^{T}{\bar{Z}}_{1}\Pi_{4}$$

$${\bar{\Phi }}_{12}=\sqrt{h_{k}}\Pi_{4}^{T}{\bar{N}}_{1}$$

$${\bar{\Phi }}_{13}=h_{U}\sqrt{h_{k}}\Pi_{4}^{T}{\bar{N}}_{2}$$

with

$${\bar{Z}}_{1}=-\text{Sym}\left[\begin{array}{ccc} {\bar{N}}_{2} & {\bar{N}}_{2} & 0 \end{array}\right]$$

$${\bar{Z}}_{2}=\text{Sym}\left[\begin{array}{ccc} {\bar{N}}_{1} & -{\bar{N}}_{1} & 2{\bar{N}}_{2} \end{array}\right]$$

$$\bar{ℜ}=\left(t-t_{k}\right){\bar{ℜ}}_{1}+{\bar{ℜ}}_{2}$$

$${\bar{ℜ}}_{1}={\bar{R}}_{1}+\frac{h_{U}^{2}}{3}{\bar{R}}_{4}-\text{Sym}\left[\begin{array}{ccc} {\bar{R}}_{5} & {\bar{R}}_{5} & 0 \end{array}\right]$$

$${\bar{ℜ}}_{2}=\text{Sym}\left[\begin{array}{ccc} {\bar{R}}_{3} & -{\bar{R}}_{3} & 2{\bar{R}}_{5} \end{array}\right]$$

$$\bar{\text{Ψ}}=\left[\begin{array}{ccccc} A_{i}G^{T} & B_{i}T_{j} & 0 & -G^{T} & B_{i}T_{j} \end{array}\right]$$

$$\bar{Q}=\left[\begin{array}{ccc} {\bar{Q}}_{1} & {\bar{Q}}_{2} & {\bar{Q}}_{3}\\ {}* & {\bar{Q}}_{4} & {\bar{Q}}_{5}\\ {}* & * & {\bar{Q}}_{6} \end{array}\right]$$

$$\bar{R}=\left[\begin{array}{ccc} {\bar{R}}_{1} & {\bar{R}}_{2} & {\bar{R}}_{3}\\ {}* & {\bar{R}}_{4} & {\bar{R}}_{5}\\ {}* & * & {\bar{R}}_{6} \end{array}\right]$$

The gain matrix *K*_*j*_ of the sampled-data controller with state quantization is defined as $K_{j}=T_{j}G^{-T}$.

Proof: Define

$$\begin{array}{lll} G & = & G_{1}^{-1},\;G_{2}=\varepsilon G_{1},\;\bar{P}=GPG^{T},\;{\bar{X}}_{2}=GX_{2}G^{T}\\ \bar{R} & = & \text{diag}\left\{G,G,G,G,G,G,G\right\}R\text{diag}{\left\{G,G,G,G,G,G,G\right\}}^{T}\\ {\bar{N}}_{2} & = & \text{diag}\left\{G,G,G\right\}N_{2}G^{T},\;{\bar{N}}_{1}=\text{diag}\left\{G,G,G\right\}N_{1}G^{T},\\ \bar{D} & = & GDG^{T}\\ \bar{Q} & = & \text{diag}\left\{G,G,G\right\}Q\text{diag}{\left\{G,G,G\right\}}^{T},\\ {\bar{X}}_{1} & = & \text{diag}\left\{G,G,G\right\}X_{1}\text{diag}{\left\{G,G,G\right\}}^{T} \end{array}$$

Equation (8) is pre- and post-multiplied by diag{*G, G, G, G, G*} and its transpose, respectively. Equation (9) is pre- and post-multiplied by diag{*G, G, G, G, G, G, G*} and its transpose, respectively. We accordingly obtain Equations (18) and (19). This completes the proof.

**Remark 3**. Theorems 1 and 2 provide the stability condition and the controller design for the sampled-data T-S fuzzy system with the quantized state, using the discontinuous LKF approach. If we do not consider the impact of the state quantization in system (Equation 7), *f*(*x*(*t*_*k*_)) in *V*_2_(*t*) could be eliminated, and the result can be degenerated for the case without the quantized state, which will be discussed in the next section.

**Remark 4**. In order to obtain less conservativeness sufficient stability condition for sampled-data T-S fuzzy systems with state quantization, some other methods combined with free-matrix-based integral inequality bounds processing technique could be used, such as parameter-dependent LKF and two-sided looped-functional.

### 3.2. Stability Analysis of Sampled-Data T-S Fuzzy System Without State Quantization

In this section, the impact of the quantized state is not considered, and the T-S fuzzy sampled-data system then becomes

(20) $$\begin{array}{r} ẋ\left(t\right)=\overset{r}{\underset{i=1}{\sum }}\overset{r}{\underset{j=1}{\sum }}h_{i}\left(\theta \left(t\right)\right)h_{j}\left(\theta \left(t_{k}\right)\right)\left[A_{i}x\left(t\right)+B_{i}K_{j}\left(x\left(t_{k}\right)\right)\right] \end{array}$$

Define

$$\begin{array}{lll} {ẽ}_{i} & = & \left[\begin{array}{ccc} 0_{n\times \left(i-1\right)n} & I_{n} & 0_{n\times \left(4-i\right)n} \end{array}\right],\\ {\tilde{\xi }}^{T}\left(t\right) & = & {\left[\begin{array}{cccc} x^{T}\left(t\right) & x^{T}\left(t_{k}\right) & \int_{t_{k}}^{t}x^{T}\left(s\right)ds & {ẋ}^{T}\left(t\right) \end{array}\right]}^{T} \end{array}$$

$${\tilde{\eta }}_{1}^{T}\left(t\right)=\left[\begin{array}{cc} x^{T}\left(t\right)-x^{T}\left(t_{k}\right) & \int_{t_{k}}^{t}x^{T}\left(s\right)ds \end{array}\right]$$

$${\tilde{\eta }}_{2}^{T}\left(t\right)=\left[\begin{array}{cc} x^{T}\left(t_{k}\right) & {ẋ}^{T}\left(t\right) \end{array}\right]$$

$${\tilde{\eta }}_{3}^{T}\left(t\right)=\left[\begin{array}{cc} {ẋ}^{T}\left(t\right) & x^{T}\left(t\right) \end{array}\right]$$

$${\tilde{\eta }}_{4}^{T}\left(t\right)=\left[\begin{array}{ccc} x^{T}\left(t\right) & x^{T}\left(t_{k}\right) & \int_{t_{k}}^{t}x^{T}\left(s\right)ds \end{array}\right]$$

$${\tilde{\eta }}_{5}^{T}\left(t\right)=\left[\begin{array}{ccc} {ẋ}^{T}\left(t\right) & 0 & x^{T}\left(t\right) \end{array}\right]$$

$${\tilde{\Pi }}_{1}^{T}=\left[\begin{array}{cc} {ẽ}_{1}^{T}-{ẽ}_{2}^{T} & {ẽ}_{3}^{T} \end{array}\right]$$

$${\tilde{\Pi }}_{2}^{T}=\left[\begin{array}{cc} {ẽ}_{2}^{T} & {ẽ}_{4}^{T} \end{array}\right]$$

$${\tilde{\Pi }}_{3}^{T}=\left[\begin{array}{cc} {ẽ}_{4}^{T} & {ẽ}_{1}^{T} \end{array}\right]$$

$${\tilde{\Pi }}_{4}^{T}=\left[\begin{array}{ccc} {ẽ}_{2}^{T} & {ẽ}_{2}^{T} & {ẽ}_{3}^{T} \end{array}\right]$$

$${\tilde{\Pi }}_{5}^{T}=\left[\begin{array}{ccc} {ẽ}_{4}^{T} & 0 & {ẽ}_{1}^{T} \end{array}\right]$$

Now, using the same method used in Theorem 1, we have the following Corollary without considering the state quantization.

**Corollary 1**. For given scalars *h*_*L*_ and *h*_*U*_ with 0 ≤ *h*_*L*_ ≤ *h*_*U*_, the system (Equation 20) is asymptotically stable, if there are some symmetric positive definite matrices $P\in S_{+}^{n}$, *X*_1_, $\tilde{Q}\in S_{+}^{2n}$, $R\in S_{+}^{7n}$ and any metrics *G*_1_, *G*_2_, *X*_2_, *N*_1_, *N*_2_ with appropriate dimensions, and for any *i* ≥ 1 and *j* ≤ *r*, the following LMIs hold

(21) $$\begin{array}{r} \Upsilon_{1}+h_{k}\Upsilon_{2}<0 \end{array}$$

(22) $$\begin{array}{r} \left[\begin{array}{ccc} \Upsilon_{1}+h_{k}\Upsilon_{3} & \Upsilon_{12} & \Upsilon_{13}\\ {}* & -{\tilde{Q}}_{3} & 0\\ {}* & * & -3{\tilde{Q}}_{3} \end{array}\right]<0 \end{array}$$

where

$$\begin{array}{l} \Upsilon_{1}=\text{Sym}\left({ẽ}_{1}^{T}P{ẽ}_{4}\right)-{\tilde{\Pi }}_{1}^{T}X_{1}{\tilde{\Pi }}_{1}-\text{Sym}\left({ẽ}_{3}^{T}X_{2}{ẽ}_{2}\right)\\ -\text{Sym}\left(\left({ẽ}_{2}^{T}{\tilde{Q}}_{2}\right)\left({ẽ}_{1}-{ẽ}_{2}\right)\right)+\Pi_{4}^{T}{ℜ}_{1}\Pi_{4}\\ +\text{Sym}\left(\Pi_{4}^{T}{ℜ}_{2}\Pi_{5}\right)+{ẽ}_{4}^{T}R_{6}{ẽ}_{4}+\Pi_{4}^{T}Z_{2}\Pi_{4}\\ +\text{Sym}\left(\left({ẽ}_{1}^{T}G_{1}+{ẽ}_{4}^{T}G_{2}\right)\tilde{\text{Ψ}}\right) \end{array}$$

$$\Upsilon_{2}=\text{Sym}\left({\tilde{\Pi }}_{1}^{T}X_{1}{\tilde{\Pi }}_{3}\right)+\text{Sym}\left({ẽ}_{1}^{T}X_{2}{ẽ}_{2}\right)+{\tilde{\Pi }}_{2}^{T}\tilde{Q}{\tilde{\Pi }}_{2}$$

$$\Upsilon_{3}=-{ẽ}_{2}^{T}{\tilde{Q}}_{1}{ẽ}_{2}+\text{Sym}\left(\Pi_{4}^{T}{ℜ}_{1}\Pi_{5}\right)+\Pi_{4}^{T}Z_{1}\Pi_{4}$$

$$\Upsilon_{12}=\sqrt{h_{k}}\Pi_{4}^{T}N_{1}$$

$$\Upsilon_{13}=h_{U}\sqrt{h_{k}}\Pi_{4}^{T}N_{2}$$

with

$$\tilde{\text{Ψ}}=\left[\begin{array}{cccc} A_{i} & B_{i}K_{j} & 0 & -I \end{array}\right]$$

$$\tilde{Q}=\left[\begin{array}{cc} {\tilde{Q}}_{1} & {\tilde{Q}}_{2}\\ {}* & {\tilde{Q}}_{3} \end{array}\right]$$

where *Z*_1_, *Z*_2_, *R*, ℜ_1_ and ℜ_2_ have been defined in Theorem 1.

Furthermore, the following Corollary regarding the sampled-data controllers design can be derived using a similar method used in Theorem 2.

**Corollary 2**. For given scalars *h*_*L*_ and *h*_*U*_ with 0 ≤ *h*_*L*_ ≤ *h*_*U*_, system (Equation 20) is asymptotically stable, if there exist symmetric positive definite matrices $\bar{P}\in S_{+}^{n}$, ${\bar{X}}_{1}$, $\hat{Q}\in S_{+}^{2n}$, $\bar{R}\in S_{+}^{7n}$ and any matrix *G*, *T*_*j*_, ${\bar{X}}_{2}$, ${\bar{N}}_{1}$, ${\bar{N}}_{2}$ with appropriate dimensions, and for any 1 ≤ *i*, and *j* ≤ *r*, the following LMIs hold

(23) $$\begin{array}{r} {\bar{\Upsilon }}_{1}+h_{k}{\bar{\Upsilon }}_{2}<0 \end{array}$$

(24) $$\begin{array}{r} \left[\begin{array}{ccc} {\bar{\Upsilon }}_{1}+h_{k}{\bar{\Upsilon }}_{3} & {\bar{\Upsilon }}_{12} & {\bar{\Upsilon }}_{13}\\ {}* & -{\hat{Q}}_{3} & 0\\ {}* & * & -3{\hat{Q}}_{3} \end{array}\right]<0 \end{array}$$

where

$$\begin{array}{l} {\bar{\Upsilon }}_{1}=\text{Sym}\left({ẽ}_{1}^{T}\bar{P}{ẽ}_{4}\right)-{\tilde{\Pi }}_{1}^{T}{\bar{X}}_{1}{\tilde{\Pi }}_{1}-\text{Sym}\left({ẽ}_{3}^{T}{\bar{X}}_{2}{ẽ}_{2}\right)\\ -\text{Sym}\left(\left({ẽ}_{2}^{T}{\hat{Q}}_{2}\right)\left({ẽ}_{1}-{ẽ}_{2}\right)\right)+\Pi_{4}^{T}{\bar{ℜ}}_{1}\Pi_{4}\\ +\text{Sym}\left(\Pi_{4}^{T}{\bar{ℜ}}_{2}\Pi_{5}\right)+{ẽ}_{4}^{T}{\bar{R}}_{6}{ẽ}_{4}+\Pi_{4}^{T}{\bar{Z}}_{2}\Pi_{4}\\ +\text{Sym}\left(\left({ẽ}_{1}^{T}+\varepsilon {ẽ}_{4}^{T}\right)\overset{}{\text{Ψ}}\right) \end{array}$$

$${\bar{\Upsilon }}_{2}=\text{Sym}\left({\tilde{\Pi }}_{1}^{T}{\bar{X}}_{1}{\tilde{\Pi }}_{3}\right)+\text{Sym}\left({ẽ}_{1}^{T}{\bar{X}}_{2}{ẽ}_{2}\right)+{\tilde{\Pi }}_{2}^{T}\hat{Q}{\tilde{\Pi }}_{2}$$

$${\bar{\Upsilon }}_{3}=-{ẽ}_{2}^{T}{\hat{Q}}_{1}{ẽ}_{2}+\text{Sym}\left(\Pi_{4}^{T}{\bar{ℜ}}_{1}\Pi_{5}\right)+\Pi_{4}^{T}{\bar{Z}}_{1}\Pi_{4}$$

$${\bar{\Upsilon }}_{12}=\sqrt{h_{k}}\Pi_{4}^{T}{\bar{N}}_{1}$$

$${\bar{\Upsilon }}_{13}=h_{U}\sqrt{h_{k}}\Pi_{4}^{T}{\bar{N}}_{2}$$

with

$$\overset{}{\text{Ψ}}=\left[\begin{array}{cccc} A_{i}G^{T} & B_{i}T_{j} & 0 & -G^{T} \end{array}\right]$$

$$\hat{Q}=\text{diag}\left\{G,G\right\}\tilde{Q}\text{diag}{\left\{G,G\right\}}^{T}$$

where ${\bar{Z}}_{1}$, ${\bar{Z}}_{2}$, ${\bar{X}}_{1}$, ${\bar{X}}_{2}$, ${\bar{N}}_{1}$, ${\bar{N}}_{2}$, *R*, ${\bar{ℜ}}_{1}$ and ${\bar{ℜ}}_{2}$ have been defined in Theorem 2, and the gain matrix *K*_*j*_ of the sampled-data controller without state quantization is defined as $K_{j}=T_{j}G^{-T}$.

**Remark 5**. The stability of the sampled-data T-S fuzzy system with and without the quantized state is investigated in Theorem 1 and Corollary 1. And the upper bound of the sampling interval for T-S fuzzy system is larger than the existing results in Lam et al. (2007), Zhu et al. (2012), Wu et al. (2014b), and Liu et al. (2016), which will be proven in further simulation examples.

## 4. Numerical Examples

This section provides two numerical examples to demonstrate the effectiveness and superiority of the proposed method.

**Example 1**. The Lorenz system with an input term (Wu et al., 2014b) is given as follows

(25) $$\left\{ \begin{array}{l} {\dot{x}}_{1}(t)=-ax_{1}(t)+ax_{2}(t)+u_{1}(t)\\ {\dot{x}}_{2}(t)=cx_{1}(t)-x_{2}(t)-x_{1}(t)x_{3}(t)\\ {\dot{x}}_{3}(t)=x_{1}(t)x_{2}(t)-bx_{3}(t) \end{array}\right.$$

The Lorenz system (Equation 25) can be represented as a type of T-S fuzzy system (Equation 2) with the following parameters

$$A_{1}=\left[\begin{array}{ccc} -a & a & 0\\ c & -1 & -d\\ 0 & d & -b \end{array}\right],\;A_{2}=\left[\begin{array}{ccc} -a & a & 0\\ c & -1 & d\\ 0 & -d & -b \end{array}\right],\;B_{1}=B_{2}=\left[\begin{array}{c} 1\\ 0\\ 0 \end{array}\right]$$

and the membership functions satisfy $h_{1}\left(x_{1}\left(t\right)\right)=\left(1+\frac{x_{1}\left(t\right)}{2}\right)/2$ and *h*_2_(*x*_1_(*t*)) = 1 − *h*_1_(*x*_1_(*t*)).

Here we choose *a* = 10, *b* = 8/3, *c* = 28, and *d* = 25.

Case I: For the case with the quantized state, take the quantizer densities as

$$\rho_{i}=1/2,\;i=1,2,3,$$

and the quantizer parameter is supposed as

$$l_{m}=\left(1-\rho_{i}\right)/\left(1+\rho_{i}\right)=1/3$$

Considering the quantized state based on Theorem 2, when ε = 0.1, the allowable maximum sampling period that can ensure the asymptotic stability of system (Equation 1) is 0.0742, which is larger than 0.0503 obtained in Liu et al. (2016) actually, and the corresponding gain matrices are

$$K_{1}=\left[-11.0256-15.249511.5024\right]$$

$$K_{2}=\left[-11.0256-15.2495-11.5024\right]$$

**Figure 1:**
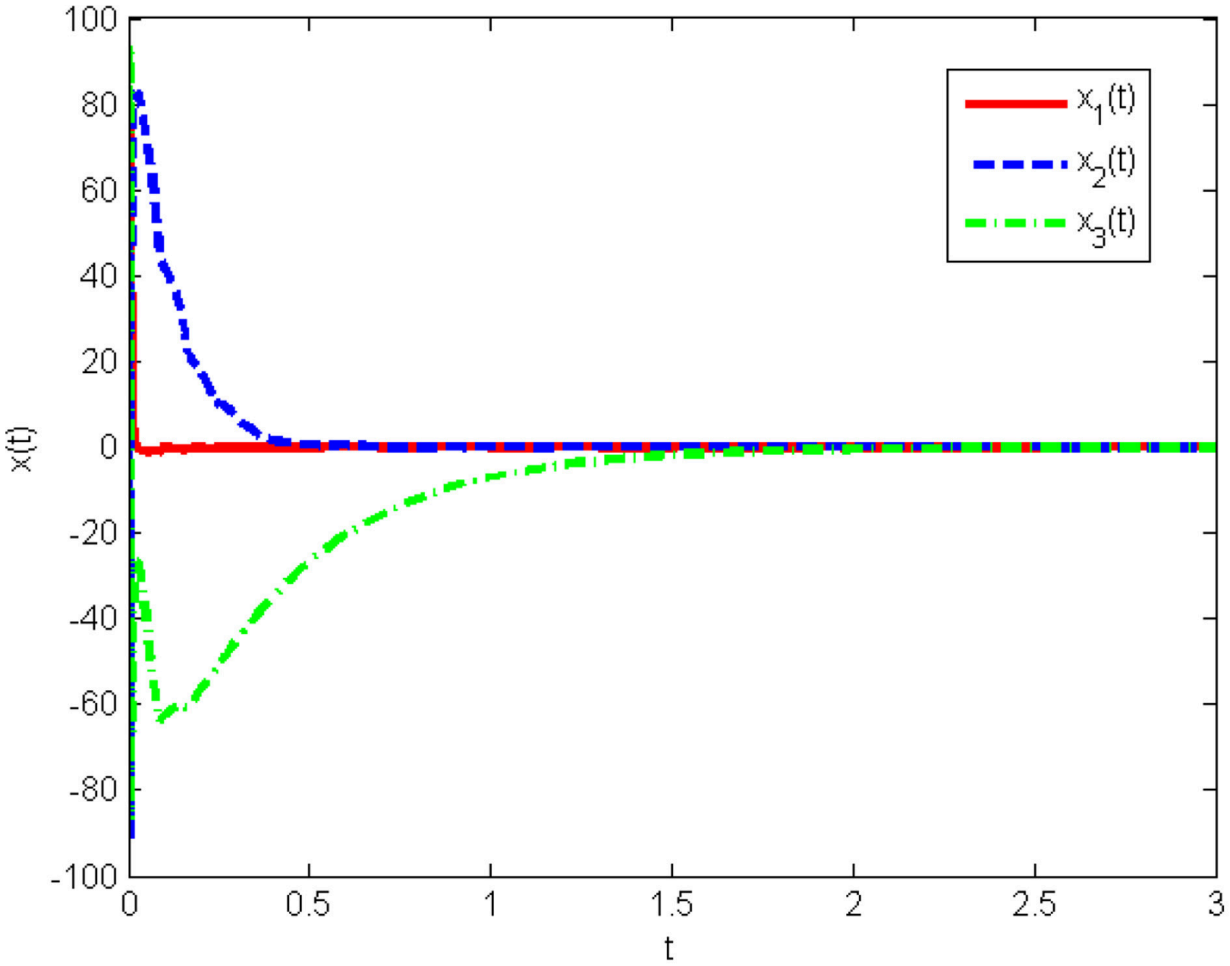
State responses of system (Equation 25) with state quantization in Example 1.

**Figure 2:**
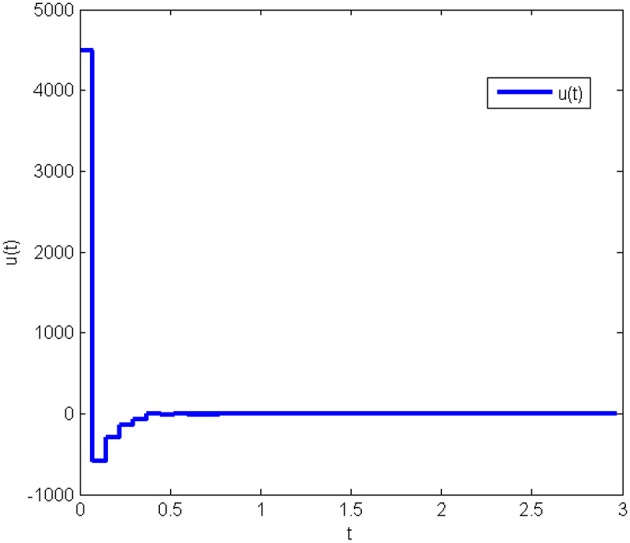
Control input of system (Equation 25) with state quantization in Example 1.

The response curves of system (Equation 25) under the initial condition *x*(0) = [20 50 80]^*T*^ with the obtained gain matrices are given in Figure 1, and the control input *u*(*t*) is shown in Figure 2. This proves that the Lorenz system (Equation 25) with state quantization under the obtained sampled-data controller, in this work, is asymptotically stable.

Case II: For the case without state quantization, based on Corollary 2, as ε = 0.1, the allowable maximum sampling period ensuring the asymptotic stability of system (Equation 7) is 0.0741. And the allowable upper bounds *h* for the sampling interval are obtained, listed in Table 1. It can be seen that the upper bound for the sampling interval under the proposed method is larger than those obtained with existing methods in Lam et al. (2007), Zhu et al. (2012), Liu et al. (2016), and Wu et al. (2014b).

**Table 1:** Maximum allowable bounds *h* without state quantization in Example 1.

	Lam et al., 2007	Zhu et al., 2012	Wu et al., 2014b	Liu et al., 2016	Corollary 2
*h*	0.0158	0.0270	0.0347	0.0560	0.0741

Simulation results are provided to verify the effectiveness of the proposed method. When *h* = 0.0741, by solving LMIs (Equations 23, 24), we can obtain corresponding gain matrices as follows

$$K_{1}=\left[-11.2795-15.291011.4783\right]$$

$$K_{2}=\left[-11.2795-15.2910-11.4783\right]$$

**Figure 3:**
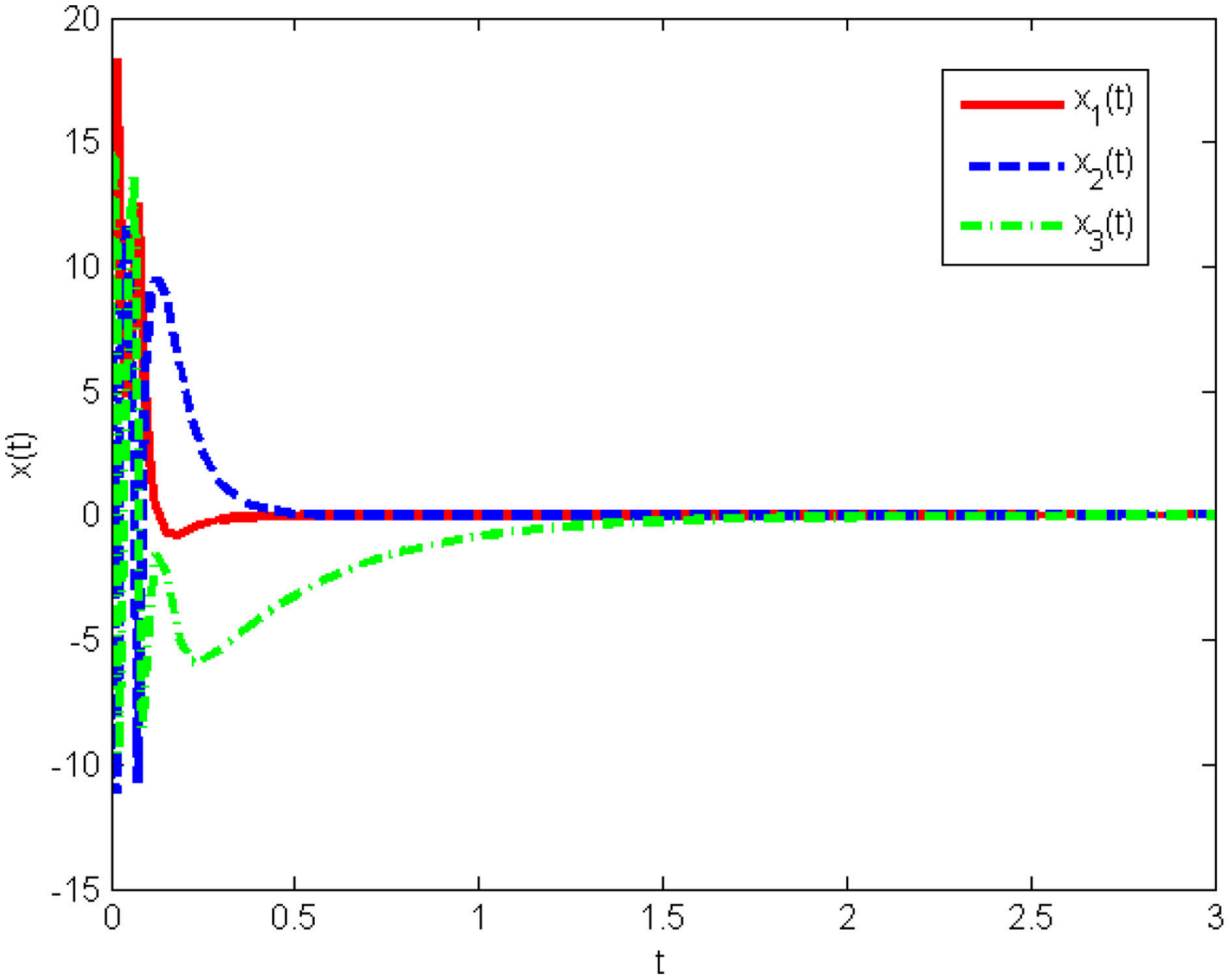
State responses of system (Equation 25) without state quantization in Example 1.

**Figure 4:**
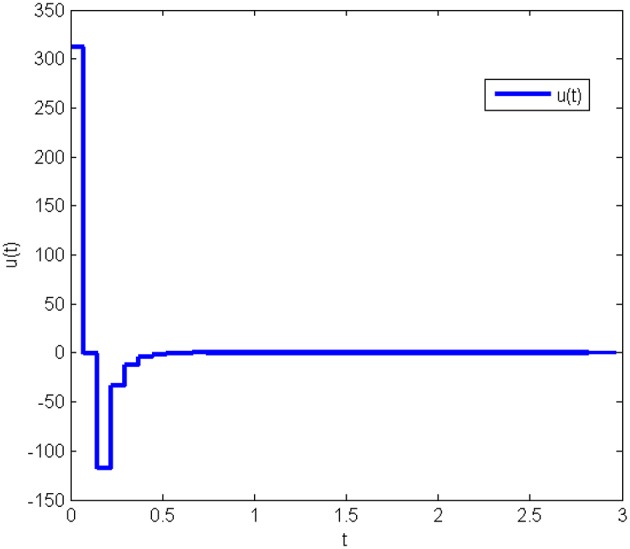
Control input of system (Equation 25) without state quantization in Example 1.

The response curves of the system (Equation 25) with the initial condition *x*(0) = [10 10 10]^*T*^ under the obtained gain matrices are given in Figures 3, 4 shows the control input *u*(*t*). This proves that the controller obtained in this work is correct and valid.

**Example 2**. The dynamic of unified chaotic system with an input term (Liu et al., 2016) is given as

(26) $$\left\{ \begin{array}{l} {\dot{x}}_{1}(t)=-(25a+10)(x_{1}(t)-x_{2}(t))+u_{1}(t)\\ {\dot{x}}_{2}(t)=(28-3a)x_{1}(t)+(29a-1)x_{2}(t)-x_{1}(t)x_{3}(t)\\ {\dot{x}}_{3}(t)=x_{1}(t)x_{2}(t)-(8+a)x_{3}(t)/3 \end{array}\right.$$

Note that the unified chaotic system (Equation 26) with *x*_1_(*t*) ∈ [−*d, d*] can be represented in the T-S fuzzy system (Equation 2) with

$$A_{1}=\left[\begin{array}{ccc} -\left(25a+10\right) & 25a+10 & 0\\ 28-3a & 29a-1 & -d\\ 0 & d & -\left(8+a\right)/3 \end{array}\right]$$

$$A_{2}=\left[\begin{array}{ccc} -\left(25a+10\right) & 25a+10 & 0\\ 28-3a & 29a-1 & d\\ 0 & -d & -\left(8+a\right)/3 \end{array}\right]$$

$$B_{1}=B_{2}=\left[\begin{array}{c} 1\\ 0\\ 0 \end{array}\right]$$

and the membership functions are *h*_1_(*x*_1_(*t*)) = (*d* + *x*_1_(*t*))/2*d* and *h*_2_(*x*_1_(*t*)) = (*d* − *x*_1_(*t*))/2*d*, respectively.

Here *a* = 0.2 and *d* = 10 are chosen, and two cases with and without state quantization are considered further.

Case I: Taking the same quantizer densities as in Example 1, and using Theorem 2, when ε = 0.01, system (Equation 26) can be asymptotically stable with the maximum sampling period *h* = 0.0535, which is larger than 0.0424 in Liu et al. (2016). Then, we choose *h* = 0.02, and by solving LMIs (Equations 18, 19), the corresponding gain matrices can be obtained as follows

$$K_{1}=\left[-25.4237-30.89792.6211\right]$$

$$K_{2}=\left[-25.4237-30.8979-2.6211\right]$$

**Figure 5:**
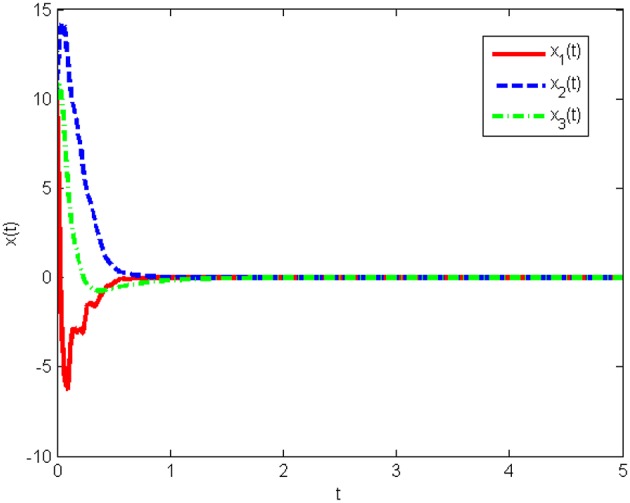
State responses of system (Equation 26) with state quantization in Example 2.

**Figure 6:**
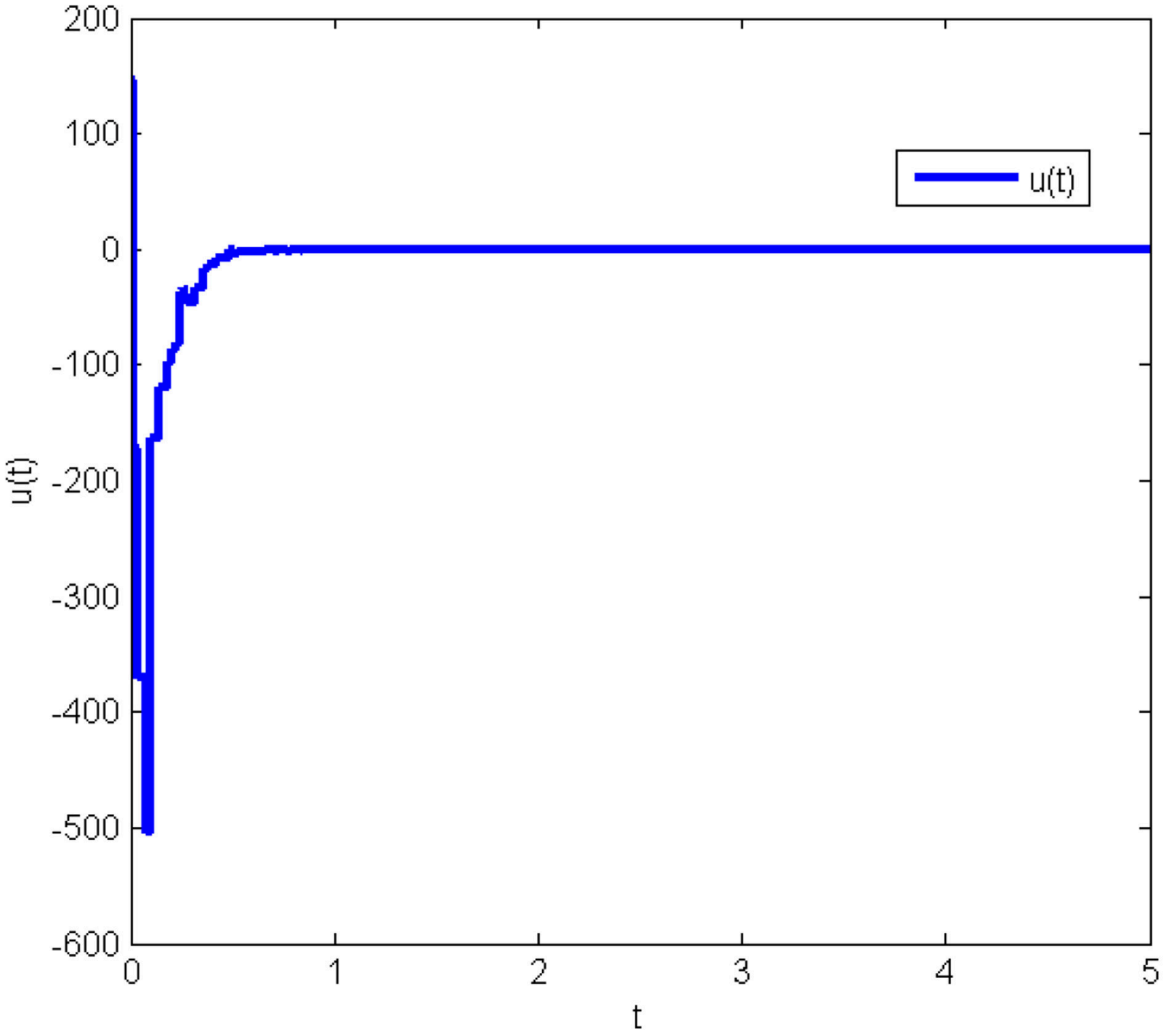
Control input of system (Equation 26) with state quantization in Example 2.

Under the obtained gain matrices and the initial condition *x*(0) = [10 10 10]^*T*^, the state response and control input *u*(*t*) for system (Equation 26) are shown in Figures 5, 6, respectively. This proves that the controller obtained in this work is correct and valid.

Case II: The maximum allowable upper bound of the sampling interval *h* without state quantization under the sampled-data control is listed in Table 2, which shows that the results in Corollary 2 is superior to existing ones.

**Table 2:** Maximum allowable bounds *h* without state quantization in Example 2.

	Zhu et al., 2012	Wu et al., 2014b	Liu et al., 2016	Corollary 2
*h*	0.0377	0.0480	0.0830	0.1040

When ε = 0.1 and *h* = 0.04, by solving LMIs (Equations 23, 24), we can obtain the corresponding gain matrices as follows

$$K_{1}=\left[-11.7978-20.67591.2851\right]$$

$$K_{2}=\left[-11.7978-20.6759-1.2851\right]$$

**Figure 7:**
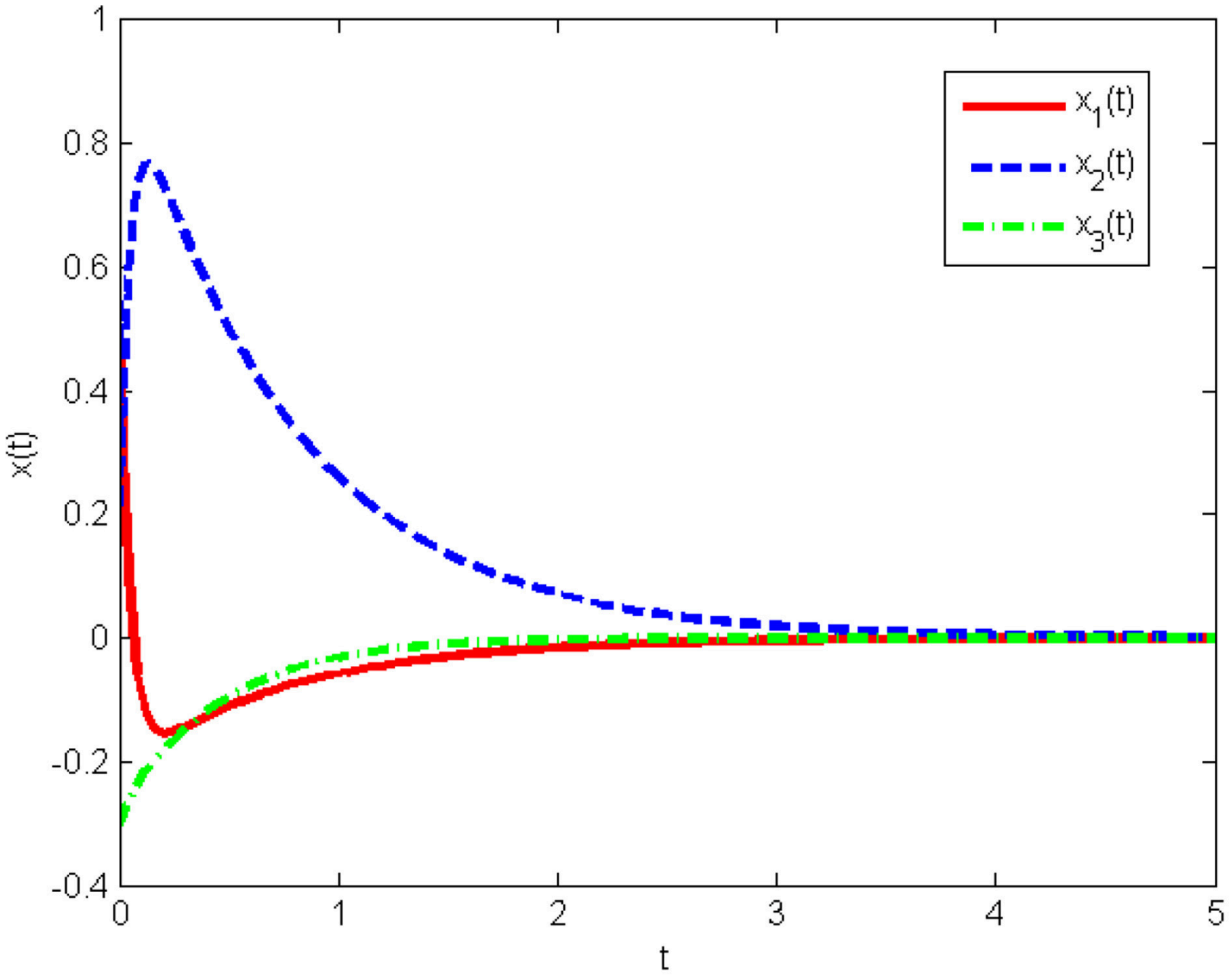
State responses of system (Equation 26) without state quantization in Example 2.

**Figure 8:**
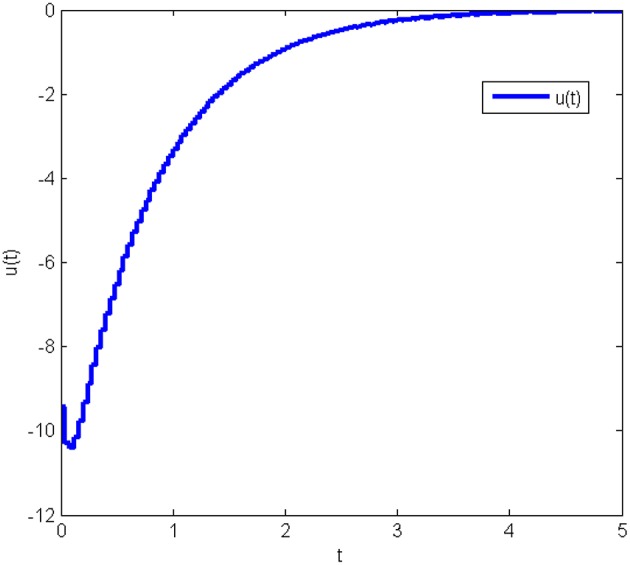
Control input of system (Equation 26) without state quantization in Example 2.

The response curves of system (Equation 26) with initial condition *x*(0) = [0.5 0.2 − 0.3]^*T*^ under the above gain matrices are displayed in Figure 7, and the corresponding control input *u*(*t*) is given in Figure 8. This proves that the controller proposed in Corollary 2 can ensure the asymptotic stability of the T-S fuzzy sampled-data system (Equation 26) without state quantization.

## 5. Conclusions

In this work, we have investigated the stability for a class of nonlinear T-S fuzzy sampled-data systems with state quantification. A new LKF approach has been constructed and a Free-Matrix-Based boundary treatment technique for integral inequalities has been adopted in order to obtain less conservative stability conditions and correspondingly, a controller has been designed. Furthermore, the stability of the T-S fuzzy sampled-data system without quantized states, has also been discussed and sampled-data controllers have been designed accordingly. The experimental results show that the maximum sampling interval for T-S fuzzy sampled-data systems with and without quantized states in our work, are both larger than the results in previous studies. Nevertheless, some other interesting problems that need to be addressed still exist, such as the reliable control design for the sampled-data T-S fuzzy systems with state quantization, and the extension of our developed approaches to the dissipativity-based sampled-data control design, which deserve further investigation.

## Author Contributions

All authors listed have made a substantial, direct and intellectual contribution to the work, and approved it for publication.

### Conflict of Interest Statement

The authors declare that the research was conducted in the absence of any commercial or financial relationships that could be construed as a potential conflict of interest.
